# Effects of Clozapine and other Atypical Antipsychotics on Infants Development Who Were Exposed to as Fetus: A Post-Hoc Analysis

**DOI:** 10.1371/journal.pone.0123373

**Published:** 2015-04-24

**Authors:** Ping Shao, Jianjun Ou, Mei Peng, Jingping Zhao, Jindong Chen, Renrong Wu

**Affiliations:** 1 Institute of Mental Health of The Second Xiangya Hospital, Central South University, 139^#^ Renmin Middle Road, Changsha 410011, Hunan, P.R. China; 2 Brains Hospital of Hunan Province, 427^#^ Renmin Middle Road (3 Duan), Changsha 410007, Hunan, P.R. China; 3 Department of Obstetrical of The Second Xiangya Hospital, Central South University, 139^#^ Renmin Middle Road,Changsha 410011, Hunan, P.R. China

## Abstract

**Objective:**

To investigate the developmental effects of clozapine and other atypical antipsychotics on infants who were exposed to as fetus.

**Method:**

The developmental progress of 33 infants who were exposed to clozapine as fetus was compared to 30 infants who were exposed to risperidone, olanzapine or quetiapine as fetus by assessing Apgar scoring, birth weight at birth, body weight, height, and the Bayley Scales of Infant and Toddler Development, Third Edition (BSID-III) at months 2, 6 and 12 of age. Five subscale scores of BSID-III including cognitive, language, motor, social-emotional, and adaptive behavior were also compared. Student’s t test and Chi-square analysis were used as appropriate. Repeated measurements were evaluated by analysis of covariance.

**Results:**

Of the 63 infants, 58 (92.1%) completed a 12-month study period. At the age of 2 and 6 months, mean adaptive behavior scores of BSID-III were significantly lower in clozapine-exposed infants than infants who exposed to other atypical antipsychotic at 2 and 6 months of age. More clozapine-exposed infants had delayed development (defined as the subscale score of <85) for adaptive behavior at 2 and 6 months of age. There was no difference between the two groups for cognitive, language, motor, social and emotional at 2, 6 and 12 months of age. More infants who were exposed to clozapine as fetus (25 of 33, 75.8%) had disturbed sleep and a labile state than those who were exposed to other atypical antipsychotics (8 of 30, 26.7%) during 2 months of age (P<0.001).

**Conclusion:**

These results suggest that clozapine has more adaptive behavior effects on infants who were exposed to as a fetus than other atypical antipsychotics at 2 and 6 months of age.

**Trial Registration:**

ClinicalTrials.gov NCT01479400

## Introduction

Female reproductive health safety and the development effect of antipsychotics on fetus has become a growing focus of concern for women who need antipsychotic treatments during their reproductive years. Majority schizophrenia women need antipsychotic treatment during the pregnancy, because non-adherence to antipsychotic treatments in patients with schizophrenia can cause relapse and poor treatment response [1–2]. It is well known that the consequence of relapse may increase personal suffering and family and societal burden [2–4].

Clozapine is very popularly used for female patients with schizophrenia in China. Usually, it is suggested that pregnant women who require treatment with clozapine should switch to one of other atypical antipsychotics because the newer atypical antipsychotics do not have some of the side effects of clozapine. But, some patients do not respond to treatment with other atypical antipsychotics. However, there is sparse data on the reproductive safety of the currently available newer compounds. Up to now, most information about atypical antipsychotics on reproductive safety and the effects on infant development come from case reports, case series, and retrospective studies [5–7]. Very few studies compared the difference effects on infants’ development for clozapine and other atypical antipsychotics.

So, there is urgent need of longitudinal prospective studies to assess the effects using atypical antipsychotics during pregnancy on infant’s development, especially neurodevelopment. Because, the prospective study was undertaken to evaluate the effect of clozapine and other atypical antipsychotics on infant development including neurobehavioral development after mothers were treated with atypical antipsychotics throughout their pregnancy. The progress of neurobehavioral development was evaluated by the Bayley Scales of Infant Development (BSID), third edition (Bayley-III) [8], a widely used measure to determine infant developmental delay. This scale has the potential to provide more clinically useful information relating to early development.

The aim of this post-hoc investigation was to assess the developmental effects of clozapine and other atypical antipsychotics on infants who were exposed to as fetus.

## Methods

This study used data from our original prospective, case-controlled study of effects of prenatal exposure to atypical antipsychotics on postnatal development, which was conducted between October 2007 and December 2010.

## Participants

Schizophrenia women with singleton pregnancies at more than 38 weeks’ were approached by study research assistants to inquire whether they could participate in the study when they gave birth in the department of obstetrical/gynecology of the Second Xiangya Hospital, Central South University, China, between October 2007 and December 2010. They were given information about the study, and if they agree to participate, they needed provide written informed consent. All enrolled pregnancy women completed a detailed questionnaire with questions regarding demographic characteristics, history of antipsychotic treatment and pregnancy outcomes which were gotten from participants, family members and medical records.

Women were assessed for schizophrenia by using the Structured Clinical Interview of DSM-IV Axis I Disorders [9], Clinical Version and were diagnosed in accordance with criteria set forth in the Diagnostic and Statistical Manual of Mental Disorders-Fourth Edition (DSM-IV) [10] during screening. Women who were identified as schizophrenia and having taking a targeted antipsychotic agent- clozapine, olanzapine, risperidone, or quetiapine alone throughout the pregnancy were recruited to be the exposed group. All expectant mothers were excluded from the study if they had evidence of liver or renal dysfunction, diabetes mellitus or cardiovascular diseases during their pregnancy.

The study was approved by the ethics committee of the Second Xiangya Hospital, and all participants provided written informed consent in accordance with National Health and Medical Research Council guidelines.

## Measures

All infants were followed for 12 months after birth. Baseline assessments included demographics and a comprehensive medical history for the mother and the birth weight, height and Apgar score at 1 and 5 minutes after birth [11] for the newborn. The assessment of infant development included body weight, height, and neurobehavioral development measurements at 2, 6 and 12 months of age.

The Bayley-III including cognitive, language, motor, social-emotional, and adaptive-behavior domains may discriminate specific development deficits caused by antipsychotics. The cognitive scale evaluates abilities, such as sensorimotor development, exploration and manipulation, object relatedness, concept formation, memory, and simple problem solving. The language scale is a composite of receptive communication (verbal comprehension, vocabulary) and expressive communication (babbling, gesturing, and utterances), and the motor scale evaluates both gross and fine motor functioning.

The Bayley-III was assessed by one psychologist who is blind to the status of the child. Parent-report questionnaires were incorporated into the Bayley-III to assess social-emotional and adaptive behavior. The raw scores for each of the scales were standardized to a mean of 100 with an SD of 15 (range, 50–150). The standardized scores were also classified into the categories of accelerated performance (>115), within normal limits (85–115), and delayed performance (<85).

The primary outcome was the difference of cognitive score between the infants who exposed to clozapine or other atypical antipsychotics. The major secondary outcomes included the differences of birth weight, height, and the percentage of developmental delay and scores of language, motor, social-emotional, adaptive-behavior between the two groups.

## Statistical Analysis

All analyses were conducted using the Statistical Package for Social Sciences, version 11.5 (SPSS Inc, Chicago, Illinois). Continuous variables were described by summary statistics such as means and standard deviations. Categorical variables were described by using frequencies and percentages. Student’s *t* test and Chi-square analysis were used as appropriate to evaluate between-group differences in baseline characteristics and changes in the scale scores from baseline to endpoint. The main strategy involved analysis of covariance (ANCOVA) with repeated measurements with corresponding baseline values as covariates. Analyses designed to compare groups were performed for all enrolled infants (Intent-To-Treat (ITT) analyses using the last observation carried forward method for missing data). The difference was considered statistically significant if a 2-tailed *p* value was less than 0.05.

## Results

### Maternal characteristics and pregnancy outcomes

As shown in Table 1, 63 newborns enrolled in this study. No infant had malformation. For all infants’ mothers, they received the treatment of clozapine (n = 33), risperidone (n = 16), olanzapine (n = 8), and quetiapine (n = 6) alone without using any benzodiazepines and mood stabilizers during the pregnancy. The duration of their illness and antipsychotic treatment before pregnancy were significantly longer in the clozapine group than the other atypical antipsychotic group (3.8 vs. 2.7 years and 2.8 vs. 1.9 years, *P*<0.001, respectively). Moreover, 54.5% of clozapine women had a BMI>23.9kg/m^2^ (the normal range, 18.5–23.9 Moreover, 54.5% of clozapine women had a BMI>23.9kg/m^2^ (the normal range, 18.5–23.9 kg/m^2^), compared with 26.7% of the other atypical antipsychotic women in pre-pregnancy (*P* = 0.025).

**Table 1:** Comparison of maternal characteristics and pregnancy outcomes between clozapine group and other atypical antipsychotics group.

Characteristic	Clozapine group (n = 33)	Other atypical antipsychotics group (n = 30)	Test statistics	*P* value
Age, mean (SD), y	31.5 (4.9)	29.8 (3.7)	1.654	0.078
Gestational age at birth, mean (SD), week	39.0 (0.5)	38.9 (0.5)	0.795	0.430
Duration of illness, mean (SD), y	3.8 (0.8)	2.7 (1.0)	4.964	<0.001
Duration of antipsychotic treatment, mean (SD), y	2.8 (0.7)	1.9 (0.6)	5.012	<0.001
Smoking during pregnancy	1 (3.0)	1 (3.3)	0.005	0.945
Unplanned pregnancy, n (%)	18 (54.5)	15 (50.0)	0.130	0.718
No take vitamin or folic acid during pregnancy, n (%)	8 (24.2)	7 (23.3)	0.007	0.933
Living arrangements, n (%)			1.861	0.394
Married	29 (87.9)	29(96.7)		
Single	1(3.9)	0 (0)		
Divorced	3 (7.9)	1 (3.3)		
Occupation, n (%)			2.170	0.538
Homemaker	12 (36.4)	10 (33.3)		
Work part-time	2 (6.1)	3(10.0)		
Work full-time	3 (9.1)	6 (20.0)		
unemployed	16 (48.5)	11 (36.7)		
Body mass index >23.9 (pre-pregnancy), n (%)	18 (54.5)	8 (26.7)	5.039	0.025
Diabetes during pregnancy, n (%)	2 (6.1)	1 (3.3)	0.258	0.612
Hypertension during pregnancy, n (%)	2 (6.1)	1 (3.3)	0.258	0.612
Breast feeding more than one month, n (%)	5 (15.2)	5(16.7)	0.027	0.869
Infant male sex	16 (48.5)	15 (50.0)	0.014	0.904

Five (7.9%) mothers (3 taking clozapine, 1 taking risperidone, and 1 taking quetiapine) relapsed, and the rest were stable during the pregnancy. Only 10 mothers breastfed their infants more than one month. For the 10 breast-feeding mothers, 5 mothers took clozapine, 2 took quetiapine, 2 took olanzapine and 1 took risperidone. There were no statistical differences in other maternal characteristics such as the mean gestational age at birth, complications during delivery and the rates of neonatal complications et al. between the two groups (Table 1). The mean dosage of clozapine, risperidone, olanzapine and quetiapine were 178.03mg, 2.06mg, 7.81mg and 550.00 mg, respectively (Table 2). The medication adherence was defined as having taken more than 80% of the study drug dose prescribed for that period. In this study 93% of mothers in both groups took 80% or more of their medications without difference between the two groups throughout the pregnancy.

**Table 2:** The information for antipsychotics using.

Antipsychotics	Minimum dosage (mg)	Maximum dosage (mg)	Mean (mg)	SD (mg)
Clozapine (n = 33)	75.00	450.00	178.03	70.37
Risperidone (n = 16)	1.00	4.00	2.06	0.85
Olanzapine (n = 8)	5.0	10.00	7.81	2.48
Quetiapine (n = 6)	400.00	600.00	550.00	83.67

### Infant developments between the two groups

Of the 63 infants, all were assessed at birth and at 2 months of age, 60 infants at 6 months of age and 58 infants at 12 months of age. The ratio of female and male infant gender was not significantly different between the two groups.

#### Weight and height development

As shown in Table 3, there were not significant differences between the two groups in the Apgar score at 5 minutes after birth, the percentage of low birth weight (less than 2.5kg), as well as the weight and height at birth, 2, 6, and 12 months of age. However, the the Apgar score at 1 minutes was higher in the clozapine group than the other atypical antipsychotic group (8.6 vs. 8.3, p = 0.030).

**Table 3:** The infants developments between clozapine and other atypical antipsychotic groups^a^.

Variables	Clozapine group (n = 33)	Other atypical antipsychotics group (n = 30)	Test statistics	*P* value
Birth weight, kg	3.2 (0.7)	3.3 (0.6)	0.831	0.409
Low birth weight, n (%)	3 (9.0)	5 (16.7)	0.814	0.367
Birth height, cm	51.2 (0.8)	50.8 (1.1)	1.312	0.195
Apgar Scoring at 1 minute	8.6 (0.5)	8.3 (0.6)	2.222	0.030
Apgar Scoring at 5 minute	9.6 (0.5)	9.4 (0.5)	1.370	0.176
2 months of age				
Weight, kg	5.3 (0.7)	5.0 (0.6)	1.750	0.085
Height, cm	59.1 (1.1)	58.6 (1.4)	1.565	0.123
Cognitive scale	89.8 (5.1)	90.6 (7.7)	0.438	0.663
Language scale	93.4 (6.7)	94.8 (8.3)	0.743	0.460
Motor scale	90.8 (5.9)	90.0 (5.3)	0.542	0.590
Social-emotional scale	95.7 (10.0)	95.0 (9.1)	0.288	0.774
Adaptive behavior scale	89.1 (8.9)	96.3 (7.6)	3.419	0.001
6 months of age				
Weight, kg	8.0 (0.9)	7.9 (0.5)	0.165	0.869
Height, cm	67.5 (2.2)	67.3 (2.3)	0.382	0.704
Cognitive scale	98.2 (8.0)	98.7 (9.3)	0.209	0.835
Language scale	95.0 (5.3)	95.8 (8.2)	0.446	0.657
Motor scale	99.5 (7.9)	102.2 (9.3)	1.248	0.217
Social-emotional scale	100.2 (10.1)	99.0 (10.3)	0.472	0.638
Adaptive behavior scale	94.8 (9.9)	100.5 (6.8)	2.613	0.011
12 months of age				
Weight, kg	10.0 (0.8)	10.1 (0.6)	0.241	0.810
Height, cm	74.8 (2.2)	75.5 (2.6)	1.125	0.265
Cognitive scale	100.7 (8.5)	100.2 (7.3)	0.295	0.769
Language scale	97.4 (6.3)	96.4 (6.5)	0.616	0.540
Motor scale	101.7 (8.1)	100.9 (7.9)	0.380	0.705
Social-emotional scale	102.0 (8.5)	102.1 (10.9)	0.027	0.979
Adaptive behavior scale	98.3 (9.4)	96.3 (7.6)	0.372	0.712

^a^Data are expressed as mean (SD).

#### Neurobehavioral development of the two groups

Mean adaptive-behavior scores of Bayley-III were lower in the clozapine group than the other atypical antipsychotic group at 2 and 6 months of age, but this difference disappeared at 12 months of age. At 2, 6 and 12 months of age, mean scores of Bayley-III cognitive, motor, social-emotional and language scales did not differ between the two groups (Table 3).

More exposed clozapine infants met the criteria for delayed development (score was <85) in the adaptive-behavior domain compared with those in the other atypical antipsychotic group at 2 and 6 months of age, and this difference disappeared at 12 months of age (Table 4). However, the delayed development percentage of cognitive, language, motor and social-emotional did not differ between the two groups at 2, 6 and 12 months of age (Table 4).

**Table 4:** The proportion of infants with delayed development defined as below 85 standard scores of the Bayley-III in infants who have fetal exposure to clozapine versus those in other atypical antipsychotics group^a^.

Variables	Clozapine group (n = 33)	Other atypical antipsychotics group (n = 30)	Test statistics	*P* value
Cognitive scale				
2 months of age	6 (18.2)	5 (16.7)	0.025	0.874
6 months of age	4 (12.1)	3 (10.0)	0.072	0.789
12 months of age	4 (12.1)	2 (6.7)	0.543	0.461
Language scale				
2 months of age	6 (18.2)	4 (13.3)	0.277	0.599
6 months of age	4 (12.1)	4 (13.3)	0.021	0.885
12 months of age	4 (12.1)	4 (13.3)	0.021	0.885
Motor sacle				
2 months of age	7 (21.2)	5 (16.7)	0.211	0.646
6 months of age	4 (12.1)	3 (10.0)	0.072	0.789
12 months of age	3 (9.1)	3 (10.0)	0.015	0.902
Social-emotional scale				
2 months of age	6 (18.2)	7 (23.3)	0.255	0.614
6 months of age	6 (18.2)	6 (20.0)	0.034	0.854
12 months of age	4 (12.1)	5 (16.7)	0.265	0.607
Adaptive scale				
2 months of age	18 (54.5)	5 (16.7)	9.727	0.002
6 months of age	10 (30.3)	3 (10.0)	3.955	0.047
12 months of age	7 (21.2)	2 (6.7)	2.715	0.099

^a^Data was expressed as No. (%)

More clozapine-exposed infants (25 of 33, 75.8%) had disturbed sleep and labile state (depending on parents’ reports) than infants who were exposed to other atypical antipsychotics during 2 months of age (8 of 30, 26.7%) (χ^*2*^ = 15.182, *P*<0.001). However, the 5 breast feeding infants who were exposed to clozapine were stable and had regular living during 2 months of age. At 6 and 12 months of age, there were not any differences in disturbed sleep and labile state between the two groups.

## Discussion

In this longest perspective study for the effects of clozapine and other atypical antipsychotics on infant’s neurodevelopment, we found that more infants who exposed to clozapine as fetus had adaptive-behavior development delay at 2 and 6 months of age than those exposed to other atypical antipsychotics. Meanwhile, infants exposed to clozapine had more disturbed sleep and labile state at 2 months of age. But all these differences disappeared after 6 months of age. These findings indicated that clozapine should have more effects on the infant’s adaptive-behavior development and these effects will not last for more than 6 months of age.

Our results were consistent with some case reports and few studies [8, 12], but not others [5–7]. Mendhekar [13] reported that an infant had delayed speech acquisition until 5 years old when the mother was taking clozapine during pregnancy and breast feeding period, but in our study we did not find any difference for language development between the two groups. Also many case reports indicate that there are not any developmental problems for infants exposed to antipsychotics during pregnancy. The inconsistency might be due to the methodology used for case reports. No case reports had used a standard measurement for assessing infants’ development like Bayley-III. All previous case studies depend on parents’ reports. Our results showed that the structure of the new Bayley-III had the potential to provide more clinically useful information relating to early development, improving our capacity to discriminate specific developmental problems and helping to target early intervention programs to more specific areas of weakness, and may be a more sensitive outcome measure for clinical trials [14–15].

Meanwhile, for these infants who were exposed to clozapine as fetus, in this post-hoc analysis we found 5 breast feeding infants who were exposed to clozapine were more stable and had regular living than those without breast feeding.These findings should be related with clozapine withdrawal syndrome. The 5 breast feeding infants have not clozapine withdrawal syndrome as clozapine can be in the breast milk. Case report has found that clozapine may induce withdrawal syndromes and newborns withdrawal of clozapine can increase the risk of convulsive and hypotonia [16]. Althouigh we did not find the withdrawal syndromes for other antipsychotics. Gilad O et al [17] reported that 10% of newborns that had withdrawal of olanzapine had withdrawal symptoms. So, we recommend that the pregnant women taking clozapine should decrease their dosage of clozapine in the last two months of pregnancy to prevent withdrawal syndrome in the infants. In terms of breast feeding, no conclusions can be drawn about the risk/benefit profile of the majority of antipsychotics in breast-feeding. In general, clozapine and olanzapine should be avoided during breast-feeding, because clozapine should be considered contraindicated for its liability of inducing potential life-threatening events in the infant, and olanzapine seems to be associated with an increased risk of inducing extrapyramidal reactions in the breast-fed babies [18]. Conversely, in patients who need to continue antipsychotic therapy during breast-feeding, it is suitable to maintain the previous pharmacologic regimen, if known to be effective.

The response of the mother and fetus/neonate to antipsychotics is determined by the distribution and elimination of the antipsychotic within and between the mother and fetus. All psychotropic medications diffuse across the placenta, which exposes the fetus to some degree of risk [19]. Newport et al [20] found that olanzapine has high placental passage ratio, and followed by haloperidol, risperidone, and quetiapine. It has been shown that neonates who are exposed to olanzapine had higher tendencies to have low birth weight and admission to the neonatal intensive care unit. However, we did not find more olanzpine exposed infants had low birth weight. But we found that more clozapine exposed schizophrenic mothers had a body mass index >23.9 kg/m^2^, and longer duration of illness and antipsychotic treatment than those exposed to other atypical antipsychotics. These should be related to weight gain induced by clozapine. Moreover, clozapine is very popularly prescribed for chronic schizophrenia patients in China.

The antipsychotic dosages of mothers were lower than acute schizophrenic patients, but the same as stable patients. The choice of antipsychotic dosage should be based on the general effectiveness profile of each agent. In pregnancy, physiological changes may result in reduced plasma protein binding, an increase in the apparent volume of distribution, and more rapid metabolic and renal clearance of certain drugs [20]. Therefore the dosages of antipsychotics may need to be altered during pregnancy. We think it should be better to supervise the serum concentration of antipsychotics and change the dosage depending on the serum concentration.

As we have reported [21], when compared to control participants, the delayed neurodevelopment appeared to be short-term, suggesting that the potential “harmful” effects of atypical antipsychotics on infant’s development are in the first 12 months of life.

The study has some limitations and the first is that the sample size was small. Therefore, the data might be not representative. We only reported the neurodevelopment of 63 infants who were exposed to antipsychotics throughout their mothers’ pregnancy and it gives some evidences for clinicians when they weigh the risks and benefits of continuous antipsychotics treatment for pregnant schizophrenia women. Second, we have not evaluated the antipsychotic concentration of the placenta. So we do not know the relationship between the antipsychotic concentration and the delayed neurodevelopment of infants. Third, we only observed the neurodevelopment at 12 months of age in infants, and do not know about the neurodevelopment of infants after 1 years old. Fourth, we did not evaluate the difference between infants who were experienced fetal-exposure to antipsychotics and control infants who were not fetal-exposure to antipsychotics. Lastly, in the study we cannot find the effect of antipsychotic on the early pregnancy outcomes such as abortion. In the future, we will do some prospective studies to examine the effect of antipsychotics on pregnancy outcomes and neonatal development. A large randomized, placebo-controlled study is also warranted to support or refute these findings.
